# Effects of disorder induced by heavy-ion irradiation on (Ba_1−*x*_K_*x*_)Fe_2_As_2_ single crystals, within the three-band Eliashberg s± wave model

**DOI:** 10.1038/s41598-017-13303-5

**Published:** 2017-10-12

**Authors:** G. Ghigo, G. A. Ummarino, L. Gozzelino, R. Gerbaldo, F. Laviano, D. Torsello, T. Tamegai

**Affiliations:** 1 Politecnico di Torino, Department of Applied Science and Technology, Torino, 10129 Italy; 2 Istituto Nazionale di Fisica Nucleare, Sez. Torino, Torino, 10125 Italy; 3 National Research Nuclear University MEPhI (Moscow Engineering Physics Institute), Moskva, 115409 Russia; 4 The University of Tokyo, Department of Applied Physics, Hongo, Bunkyo-ku, Tokyo, 113-8656 Japan

## Abstract

One of the open issues concerning iron-based superconductors is whether the s± wave model is able to account for the overall effects of impurity scattering, including the low rate of decrease of the critical temperature with the impurity concentration. Here we investigate Ba_1−*x*_K_*x*_Fe_2_As_2_ crystals where disorder is introduced by Au-ion irradiation. Critical temperature, *T*_*c*_, and London penetration depth, *λ*_*L*_, were measured by a microwave resonator technique, for different values of the irradiation fluence. We compared experimental data with calculations made on the basis of the three-band Eliashberg equations, suitably accounting for the impurity scattering. We show that this approach is able to explain in a consistent way the effects of disorder both on *T*_*c*_ and on *λ*_*L*_(*T*), within the s± wave model. In particular, a change of curvature in the low-temperature *λ*_*L*_(*T*) curves for the most irradiated crystals is fairly well reproduced.

## Introduction

The s± phase^1,2^, an extended *s*-wave pairing with a sign reversal of the order parameter between different Fermi surface sheets, is the leading candidate for describing the pairing state in most of the iron based superconductors (IBS), including the BaFe_2_As_2_ family. According to this approach, superconductivity in these compounds is unconventional and mediated by antiferromagnetic spin fluctuations. However, a general consensus has not been achieved yet, also because of a number of experiments on the effects of non-magnetic impurities that apparently do not fit the s± scenario. In particular, several substitution and particle-irradiation studies reported a much weaker rate of suppression of the critical temperature as a function of the scattering rate than initially suggested for a sign-changing order parameter^3,4^. Moreover, other unconventional mechanisms have been put forth for describing IBS, e.g. the orbital fluctuations, which favors the s++ state without sign reversal^5^. A disorder-induced change from sign-reversed (s±) to sign-preserved (s++) symmetry in multi-band superconductors has been predicted^6,7^ and observed^8^. Finally, nodal-like behaviors at increasing disorder was observed, e.g. in highly substituted crystals^9^. Nevertheless, these evidences are not necessarily against the s± picture in disordered samples. As a matter of fact, numerous unexpected novel superconducting properties, including non-trivial impurity effects – that can be even mistaken as evidence for a nodal gap state – can be explained within the s± wave model. This was demonstrated by two-gap analyses^10,11^, but should be quantitatively confirmed by a comparison of experimental results to more complete models, capturing the rich and complex physics of the material. In fact, there is evidence that at least three bands are required to satisfactorily describe the physical properties of BaFeAs-based compounds^12^.

In this work, we study the effects of disorder on Ba_1−*x*_K_*x*_Fe_2_As_2_ crystals, with the aim to determine if a discrepancy exists between the disorder-induced suppression of *T*_*c*_ and the modifications of the London penetration depth (*λ*_*L*_) on one hand, and the theoretical expectation for the s± phase on the other hand. Indeed, the analysis of *λ*_*L*_(*T*) and its modifications as a function of the rate of scattering by impurities has been used to shed light on the order parameter symmetry, the presence of nodes, and whether they are accidental or symmetry-imposed. A standard scheme proposed in literature is the following^13^: in the case of a superconductor with line nodes, impurity scattering is expected to change the linear temperature dependence of the penetration depth at $T\ll {T}_{c}$ to become quadratic. On the other hand, intraband scattering would lift the c-axis line nodes in the case of an extended s-wave and induce a change from *T* ^2^ to an exponential behavior^14^. On the contrary, if the starting system is a fully-gapped superconductor, introducing pair-breaking scattering would result in a change from exponential to *T* ^2^ variation, and to lower power in the presence of accidental nodes^11^.

We tested this scheme with samples where disorder was introduced by 250-MeV Au-ion irradiation. Particle irradiation is the way to induce defects without contributing extra chage or huge structural distorsions, contrary to most cases of chemical substitutions. Several experiments have been performed with iron-based superconductors in the last years^10,15–28^, but due to the large variety of used particles and compounds, a clear picture has not been reached yet, and a systematic study is still lacking. TEM microscopy revealed that 200-MeV Au-ion irradiation of Ba_1−*x*_K_*x*_Fe_2_As_2_ crystals results in the formation of defects that are linearly correlated along the ion track but – contrary to the case of high-*T*_*c*_ cuprates – due to the metallic nature of the compound, they are discontinuous (30–240 nm in length)^27^. In fact, magneto-optical imaging showed the clear signature of anisotropic flux pinning by discontinuous tracks in crystals irradiated with Au ions at the higher energy of 2 GeV^20^, which are present also with irradiation at lower energies (250 MeV)^29^. Moreover, a significant difference with respect to cuprates emerged from a STM analysis of irradiated Fe(Se,Te) crystals, showing that columnar defects produced by 250-MeV Au-ions have a metallic core^28^, instead of the insulating core of the amorphous tracks that the same ions with the same energy produce in cuprates^30^. In addition, significant defects other than correlated tracks are produced, such as smaller and more distributed cascades, beside the effects of secondary electrons and strain.

In fact, our data on heavy-ion irradiated Ba_1−*x*_K_*x*_Fe_2_As_2_ crystals do not seem to fit any case of the simplified *λ*_*L*_(*T*) scheme described above. Thus, a more general theoretical approach is needed to describe the experimental results. Two main aspects in this regard are to be considered: the multi-band nature of the system and the effect of disorder. On one hand, Eliashberg’s theory is the optimal model to describe these systems. Within this approach and especially when the number of bands is higher than two, disorder can be conveniently treated within the Born approximation^31^, otherwise the T-matrix calculation would increase the number of parameters, making the comparison with experimental data more questionable and possibly not conclusive. Within the Born approximation, the model is sensitive to the global effect of disorder rather than to the details of defects. As a matter of fact, a more general theory to study the role of defects in superconductors was developed^32–34^, but in the proposed formulation only applies to two-band systems, even if its generalization to more bands is possible. Since there is evidence that in the system under study the inter-band coupling is predominant and the number of bands to be considered is greater than or equal to three^35,36^, and since using two-band models would lead to the appearance of intra-band terms that do not have a physical interpretation^37^, we preferred to consider three conducting bands, rather than a more precise parametrization of disorder. Being convinced that this approach is decisive for capturing the essential physics of the material, even if other aspects are unavoidably disregarded, we considered a three-band Eliashberg model, simple enough to allow a meaningful comparison with experimental results. Within this frame, the comparison led us to the awareness that the s± wave model is able to reconcile apparently different evidences, once the impurity scattering role is accounted for.

## Techniques

High-quality Ba_1−*x*_K_*x*_Fe_2_As_2_ crystals with an analyzed doping level of x = 0.42 were grown by the FeAs self-flux method^26^, and cut as thin plates with thickness (along the *c*-axis) of about 10 *μ*m, much smaller than width and length. Disorder was induced by 250-MeV Au-ion irradiation, with the ion beam parallel to the *c*-axis of the crystals.

The characterization of the crystals is carried out by a microwave superconducting resonator technique, in a cavity perturbation approach^38,39^, described in the *Methods* section, allowing us to determine the critical temperature and the penetration depth of small crystals.

Experimental data are discussed within the s± wave model in the presence of impurity scattering, by comparing them to calculations based on the solution of the three-band Eliashberg equations with suitable input conditions, as detailed below.

## Results

### Suppression and broadening of the superconducting transition

**Figure 1:**
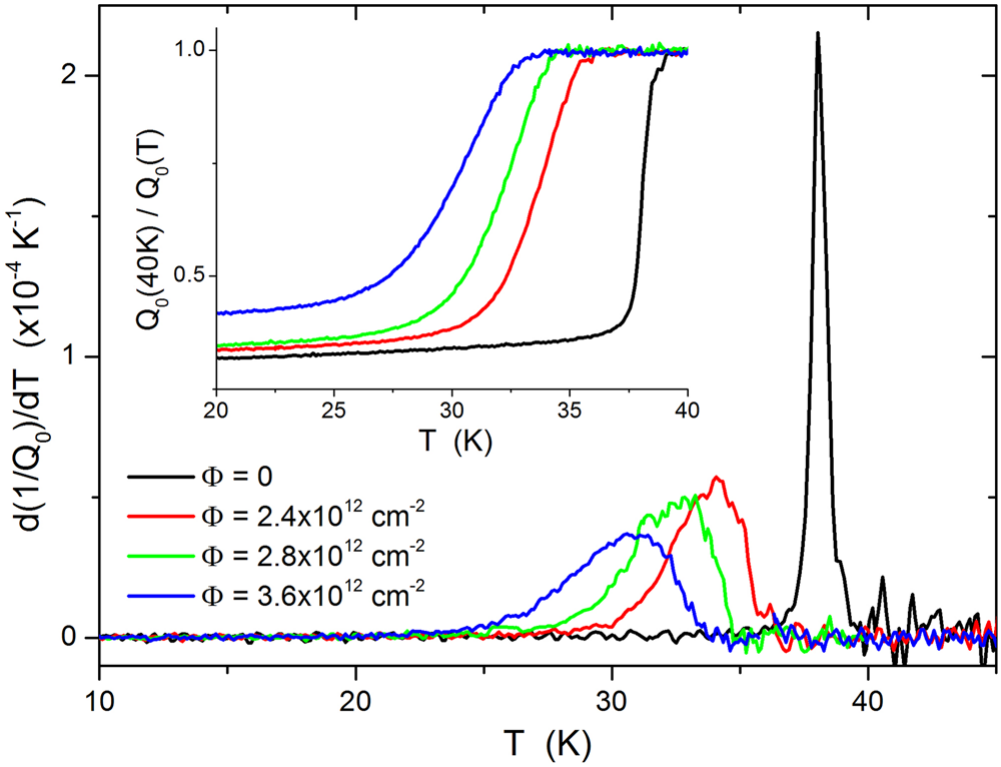
Temperature and width of the superconducting transition, from rough data. The inset shows the temperature dependence of the inverse of the resonator quality factor in the presence of the Ba_1−*x*_K_*x*_Fe_2_As_2_ sample, normalized to its value at *T* = 40 K. Data are shown for the same crystal before and after subsequent irradiations. In the mainframe, derivatives are reported, showing that irradiations cause *T*_*c*_ suppression and transition-width enlargement.

A first characterization of the superconducting transition and its modification after irradiation – in terms of temperature and width – can be obtained from rough data, without any model or analysis assumption. The inset in Fig. 1 shows the temperature dependence of the inverse of the unloaded quality factor when the Ba_1−*x*_K_*x*_Fe_2_As_2_ crystal is coupled to the resonator, normalized to its value at *T* = 40 K (see the *Methods* section for details). Since 1/*Q*_0_ is proportional to losses, such curves are qualitatively comparable to surface resistance curves. Data are shown for the same crystal before and after subsequent irradiations, up to the fluence of 3.6 × 10^12^ cm^−2^. In the mainframe, derivatives are reported, clearly showing that irradiations cause *T*_*c*_ suppression and broadening of the transition. More meaningful values of *T*_*c*_, useful for a quantitative comparison with theoretical calculations, will be obtained below after the determination of the penetration depth.

### Effects of irradiation on the penetration depth

**Figure 2:**
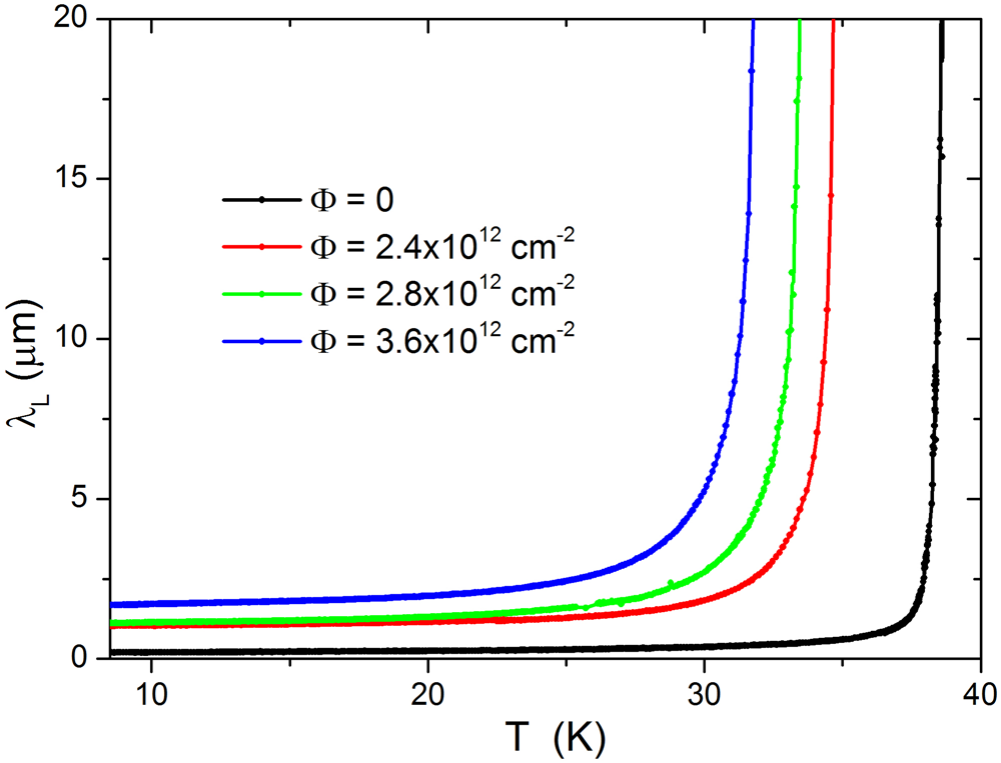
Penetration depth as a function of temperature, for the same Ba_1−*x*_K_*x*_Fe_2_As_2_ crystal, before and after subsequent irradiations up to a total fluence of Φ = 3.6 × 10^12^ cm^−2^.

**Figure 3:**
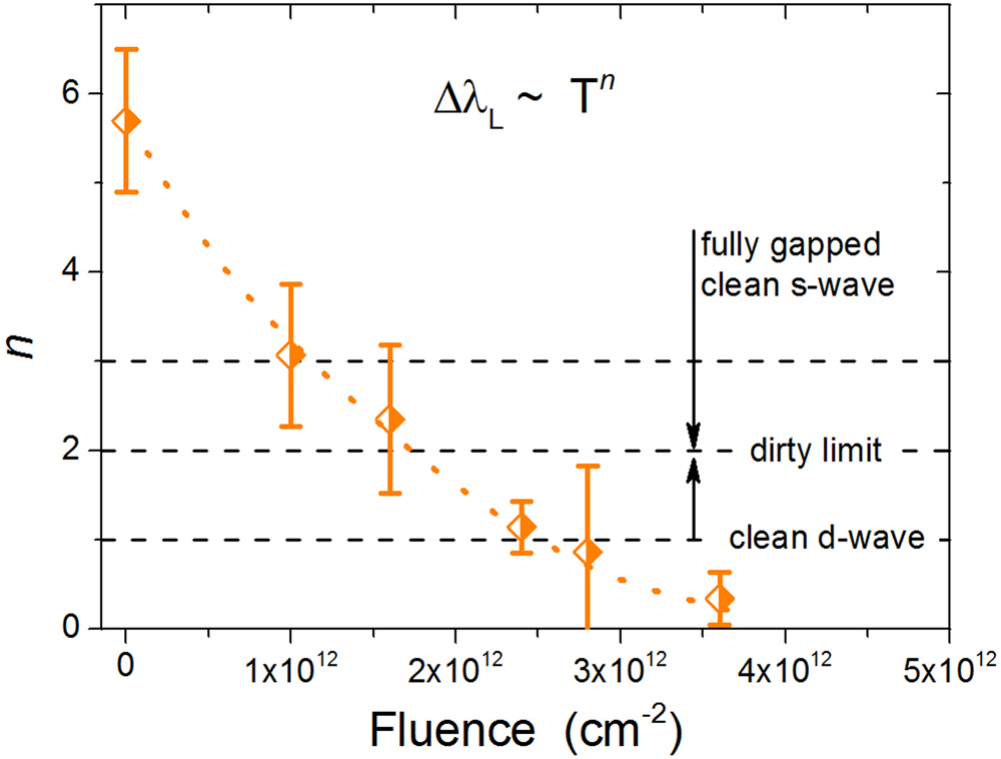
Exponent *n* of the *λ*_*L*_(*T*) power law for unirradiated and irradiated samples, as a function of the fluence. For the determination of *n*, an upper limit for the temperature range of *T*/*T*_*c*_ = 0.35 was adopted. Dashed lines set reference values (see text), while the dotted line is a guide for the eye.

Figure 2 shows the London penetration depth *λ*_*L*_ as a function of temperature, for the same crystal before and after subsequent irradiations. The effect of ion irradiation is both to enhance the *λ*_*L*_ values and to modify its temperature dependence. First of all, we investigate the low-temperature behavior of *λ*_*L*_ in order to check if it can be understood within the standard scheme described in the Introduction. Data below *T*/*T*_*c*_ = 0.35 are fitted to a power law, Δ*λ*_*L*_ ∝ *T* ^*n*^. Figure 3 reports the exponent *n* for unirradiated and irradiated samples, as a function of the fluence. Dashed lines indicate reference literature values: an exponent *n* higher than 3 can be considered to approach the exponential behavior, which is indicative of a fully-gapped clean *s*-wave state. On the other side, a *d*-wave state would imply *n* ≈ 1. In both the cases, the addition of impurity-driven scattering would lead to the dirty limit at *n* ≈ 2. This is not the trend we observed (symbols), since a smooth decrease from $n\gg 3$ to *n* < 1 is clearly shown, as the irradiation fluence is increased. It must be noted that the temperature evolution shown in Fig. 3 is not strictly speaking the low-temperature asymptotic one, that is not accessible by the experiment. Nevertheless, it gives the qualitative indication that a more rigorous theoretical approach is needed to describe the experimental data.

### Three-band Eliashberg s± calculations

To understand these results, a comparison with a theoretical model capturing the essential physics of the material is needed. We used a three-band Eliashberg s± wave model in the presence of impurity scattering, with the constraint that both the *T*_*c*_ suppression and the *λ*_*L*_(*T*) behavior were simultaneously explained. The electronic structure of the compound Ba_1−*x*_K_*x*_Fe_2_As_2_ can be approximately described by two hole bands *α*, *β* (indicated in the following as bands 1 and 2) and one equivalent electron band *γ* (indicated in the following as band 3)^40^. Within the *s*± wave model, coupling between the electron and the hole bands is due to the antiferromagnetic spin fluctuations, and the gap of the electron band, Δ_3_, has opposite sign with respect to the gaps of the hole bands, Δ_1_ and Δ_2_^1^. To calculate the gaps and the critical temperature by the three-band Eliashberg equations^41–43^ one has to solve six coupled equations for the gaps Δ_*i*_(*iω*_*n*_) and the renormalization functions *Z*_*i*_(*iω*_*n*_), where *i* is a band index ranging from 1 to 3 and *ω*_*n*_ are the Matsubara frequencies (more details in the *Methods* section). The gaps are assumed to be isotropic due to the low values of anisotropy typical of optimally doped Ba_1−*x*_K_*x*_Fe_2_As_2_ (see also the discussion below). Moreover, considering gap anisotropy would complicate greatly the equations without significantly changing the physics of this system. The calculation is based on the assumption that the nonmagnetic impurity-scattering rates ${{\rm{\Gamma }}}_{ij}^{N}$ are directly proportional to the defect density that in turn is proportional to the irradiation fluence, while ${{\rm{\Gamma }}}_{ij}^{N}$ is taken to be zero for the unirradiated crystal. In principle, this introduces a lot of new free parameters (the ${{\rm{\Gamma }}}_{ij}^{N}$ constants are proportional to the structural disorder in an unknown way). Nevertheless, since the diagonal components ${{\rm{\Gamma }}}_{ii}^{N}$ do not affect the superconducting properties, we study separately the effects of pair-breaking ${{\rm{\Gamma }}}_{12}^{N}$, ${{\rm{\Gamma }}}_{13}^{N}$, and ${{\rm{\Gamma }}}_{23}^{N}$.

**Figure 4:**
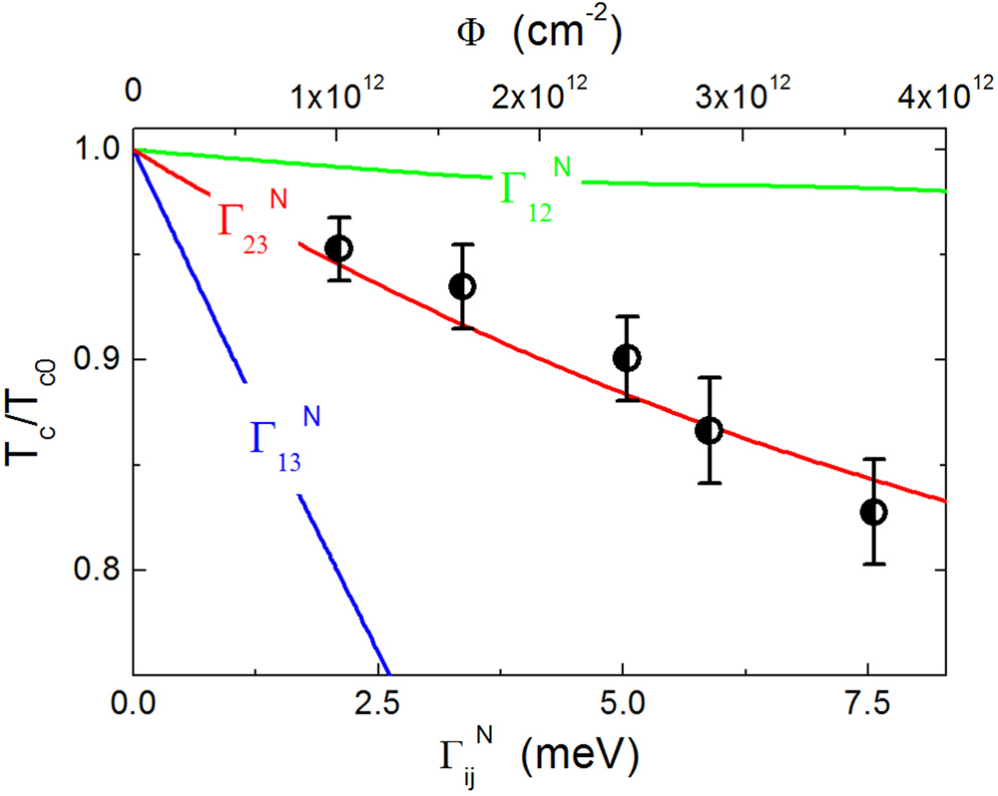
Critical temperature of the irradiated samples, *T*_*c*_, normalized by the critical temperature of the unirradiated sample, *T*_*c*0_, as a function of the interband scattering rate, ${{\rm{\Gamma }}}_{ij}^{N}$ (lower scale). The lines represent the three possible interband scattering channels, between bands 1, 2 and 3. Experimental data (symbols) are reported as a function of the fluence (upper scale). The upper and lower abscissa have been scaled until data collapsed onto one of the theoretical curves (the scaling factor is 4.8 × 10^14^ eV^−1^ cm^−2^; ${{\rm{\Gamma }}}_{23}^{N}$ was chosen for the reasons described in the text).

For the sake of simplicity, we assume that disorder mainly affects only one of the interband channels. The lines in Fig. 4 represent the solutions of the Eliashberg equations for *T*_*c*_/*T*_*c*0_ with disorder in the channels 12, 13, and 23, respectively, as a function of the scattering rate (lower scale). The experimental data (symbols) are shown as a function of the irradiation fluence (upper scale). The two scales have been adjusted to obtain a good agreement between experimental data and the ${{\rm{\Gamma }}}_{23}^{N}$ curve, giving the scale factor between the fluence and the scattering rate (4.8 × 10^14^ eV^−1^ cm^−2^, in this case). Here the *T*_*c*_ value is determined as the temperature where *λ*_*L*_(*T*) diverges. Attempts to do the same with the ${{\rm{\Gamma }}}_{12}^{N}$ and ${{\rm{\Gamma }}}_{13}^{N}$ curves were not successful, i.e. did not lead to proper *λ*_*L*_(*T*) curves. We conclude here that the main mechanism responsible for *T*_*c*_ suppression is radiation-induced increase of the interband scatting rate between bands 2 and 3. Now the energy gaps can be calculated also for the irradiated crystals.

**Figure 5:**
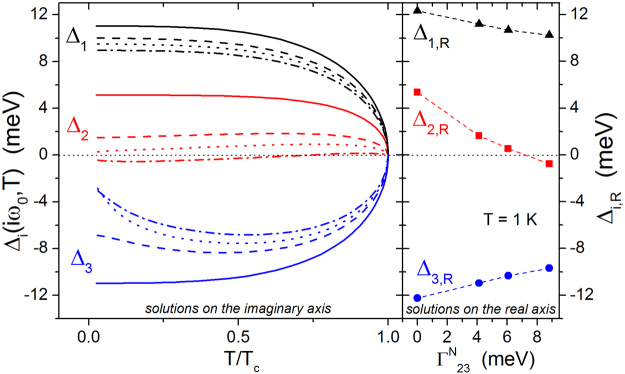
Left: temperature dependence of the first value of the energy gaps obtained by the solution of the imaginary-axis Eliashberg equations, Δ_1_ (black), Δ_2_ (red) and Δ_3_ (blue) for the unirradiated material (solid lines) and for the material with the same level of disorder as the samples irradiated at the fluences Φ shown in Fig. 2, i.e. Φ = 2.4 × 10^12^ cm^−2^ (${{\rm{\Gamma }}}_{23}^{N}$ = 4.12 meV, dashed lines), Φ = 2.8 × 10^12^ cm^−2^ (${{\rm{\Gamma }}}_{23}^{N}$ = 6.07 meV, dotted lines), and Φ = 3.6 × 10^12^ cm^−2^ (${{\rm{\Gamma }}}_{23}^{N}$ = 8.81 meV, dashed dotted lines). Right: low-temperature values of the gaps from the real-axis solutions of the Eliashberg equations, obtained both by the Padé approximants and by a direct calculation, as a function of the scattering rate (dashed lines are guides to the eye).

In Fig. 5 we report the gaps corresponding to the levels of disorder of the same sample as in Fig. 2, for each irradiation fluence (different colors for the three gaps and different line styles for the different fluences). The left-side panel shows the gap amplitudes obtained by the imaginary-axis solution of the Eliashberg equations. Remarkably, Δ_2_(*T*) for the highest fluence changes sign. The right panel of Fig. 5 shows the real-axis low-temperature gap amplitudes, as a function of the interband scattering in the 23 channel. These values, deduced both by the Padé approximants and by a direct calculation on the real axis, are slightly different from the solutions on the imaginary axis, as expected because of the strong coupling regime. For the unirradiated-crystal case, we find gap values that are in fairly good agreement with earlier literature data^40^. It is confirmed that the low-temperature Δ_2_ value for the most disordered case is negative, both on the real and on the imaginary axis. It is also shown in Fig. 5 that Δ_2_ for the most disordered case changes sign as a function of temperature. The change of sign as a function of scattering and/or temperature is worthy to be discussed in more detail.

A change of sign as a consequence of increased disorder was discussed in refs^32–34^. To qualitatively understand this behavior in our case, it is sufficient to write the Eliashberg equation for the gap Δ_2_: ${{\rm{\Delta }}}_{2}(i{\omega }_{n})\,{Z}_{2}(i{\omega }_{n})$ = ${\sum }_{m}\,[-{\lambda }_{23}(i{\omega }_{n}-i{\omega }_{m},T)+{{\rm{\Gamma }}}_{23}^{N}\,{\delta }_{{\rm{mn}}}]\,{{\rm{\Delta }}}_{3}(i{\omega }_{m},T)/\sqrt{{{\rm{\Delta }}}_{3}^{2}(i{\omega }_{m},T)+{\omega }_{m}^{2}}$, where *λ*_23_, ${{\rm{\Gamma }}}_{23}^{N}$ and *Z*_2_ are positive, while Δ_2_ is negative and *δ*_mn_ is the Kronecker delta. The presence of disorder brings to an effective coupling (the quantity in the square bracket) that can change sign, resulting in a change of sign also for Δ_2_.

A sign change as a function of temperature was discussed in ref.^44^. To understanding this particular effect it is sufficient to consider the previous equation for *n* = 0: ${{\rm{\Delta }}}_{2}(i{\omega }_{0})\,{Z}_{2}(i{\omega }_{0})$ = ${\sum }_{m}\,[-{\lambda }_{23}(i{\omega }_{0}-i{\omega }_{m},T)$ ${{\rm{\Delta }}}_{3}(i{\omega }_{m},T)/\sqrt{{{\rm{\Delta }}}_{3}^{2}(i{\omega }_{m},T)+{\omega }_{m}^{2}}]$ + ${{\rm{\Gamma }}}_{23}^{N}{{\rm{\Delta }}}_{3}(i{\omega }_{0},T)/\sqrt{{{\rm{\Delta }}}_{3}^{2}(i{\omega }_{0},T)+{\omega }_{0}^{2}}$. When the temperature increases, the value of Δ_3_(*iω*_0_, *T*) decreases in absolute value. It can happen that the positive first term overcomes the negative second term, resulting in a change from negative to positive sign for Δ_2_.

**Figure 6:**
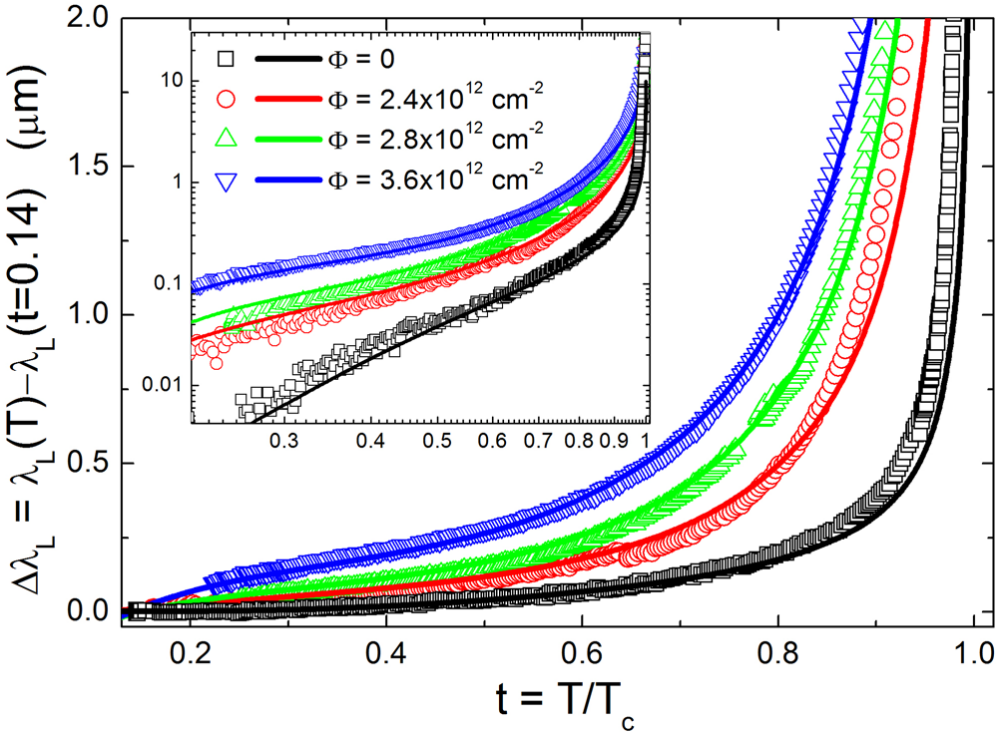
Penetration depth shift Δ*λ*_*L*_(*t*) = *λ*_*L*_(*t*) − *λ*_*L*_(*t* = 0.14), where *t* = *T*/*T*_*c*_ is the reduced temperature. Experimental data (symbols) are compared to model calculations (lines), for the unirradiated sample (Φ = 0) and for the same sample after subsequent irradiations, up to a total fluence Φ = 3.6 × 10^12^ cm^−2^. The inset shows the same data in a logarithmic scale.

Once the gaps and the main model parameters are calculated on the bases of the *T*_*c*_ behavior, the penetration depth can be computed by

1 $${\lambda }^{-2}(T)={(\frac{{\omega }_{p}}{c})}^{2}\,\sum _{i=1}^{3}\,{(\frac{{\omega }_{p,i}}{{\omega }_{p}})}^{2}\,\pi T\,\sum _{n=-\infty }^{+\infty }\,\frac{{{\rm{\Delta }}}_{i}^{2}({\omega }_{n})\,{Z}_{i}^{2}({\omega }_{n})}{{[{\omega }_{n}^{2}{Z}_{i}^{2}({\omega }_{n})+{{\rm{\Delta }}}_{i}^{2}({\omega }_{n}){Z}_{i}^{2}({\omega }_{n})]}^{\mathrm{3/2}}}$$

where *ω*_*p*,*i*_ are the plasma frequencies of the three bands and *ω*_*p*_ is the total plasma frequency. Now, we can only act on the weights of the three bands, ${(\tfrac{{\omega }_{p,i}}{{\omega }_{p}})}^{2}$ in order to adapt the calculation to the experimental *λ*_*L*_(*T*). The best results are reported in Fig. 6, where experimental Δ*λ*_*L*_(*T*) (symbols) are compared to the calculations (lines).

## Discussion and Conclusions

Iron-based superconductors display physical properties which are intimately related to their multiband structure. Since a subtle dependence of these properties on tiny impurity concentrations is expected, the investigation of the role of disorder in such compounds is crucial to achieve a comprehensive understanding. As stated in the Introduction, one of the issue still under debate is the symmetry of the superconducting order parameter, in spite of the huge efforts to conceile experiments with theoretical models. In fact, most of the analysis were done within a simplified two-band model, which is sometimes misleading. Here we have shown that a three-band approach is able to explain in a consistent way the effects of disorder both on *T*_*c*_ and on *λ*_*L*_(*T*), within the s± wave model. Figure 6 shows a remarkable agreement between experiment and theory, in particular the change of curvature in the Δ*λ*_*L*_(*T*) curves for the most irradiated cases is nicely reproduced.

Of course, the calculation still contains approximations and simplifications that could be refined. It is important to resume them and to estimate their possible influence to results. For example, we have found that the irradiation-induced scattering mainly affects the 23 interband channel: the selection of the 23 channel as the most significant follows the analysis of all the channels one by one, that is reasonable in view of a simplicity criterion. However, it cannot be excluded that other more complex combinations of the parameters could describe the experimental results as well, since it is reasonable that also the other channels are somehow modified. Moreover, we did not take into account the finite dimensions of the defects, and the presence of different kinds of defects (as stated in the Introduction, here disorder is treated in the Born approximation). The *λ*_*L*_ anisotropy was disregarded (in fact, it is among the lowest for iron-pnictides). Also the gap-anisotropy effect was not considered here. For example, we estimate that, if the smallest gap in *k* space was much smaller than the smallest average gap on one of the bands, the effect would be a decrease of the two free coupling constants (*λ*_23_ decreases in a more pronounced manner), but the critical temperature would not be affected significantly, since in this case it strongly depends on *λ*_13_. Moreover, this material has already been studied – with good results – with an isotropic model^45^ and the presence of disorder and the strong coupling regime reduce the impact of gap anisotropy^46^. Finally, due to the proximity to a magnetic state in such compounds, the possibility of also inducing magnetic scattering by defects cannot be ruled out.

However, from the point of view of computation it is very hard to suitably consider all these aspects, and the adopted model is a good compromise between the need of a reliable tool to explain experimental trends and the need of physical correctness. In this sense, the best agreement between experiment and model shown in Fig. 6 led us to the following conclusions, some of them quantitative and some other only qualitative: (i) for the unirradiated sample, the weights of the three bands 1, 2 and 3 are 0.1, 0.8 and 0.1, respectively^38^, and the total plasma frequency is *ω*_*p*_ = 1 eV, in nice agreement with ref.^47^; (ii) the weight of the band 1 is not significantly affected by irradiation, while the weight of the band 2 decreases as a function of fluence and the weight of the band 3 increases; (iii) the total plasma frequency decreases as a function of fluence, down to the value of *ω*_*p*_ = 0.14 eV for the most irradiated sample, in agreement with the observed increase of *λ*_*L*_ in irradiated samples; (iv) also a sub-linear *λ*_*L*_ temperature dependence is fully consistent with the s± wave model, if the effects of impurity scattering is suitably taken into account.

## Methods

### Ion irradiation

Irradiations were performed at room temperature at the Tandem-XTU facility of the LNL laboratories of the Italian National Institute for Nuclear Physics (INFN). The ion beam was parallel to the *c*-axis of the crystals, and to minimize the heating of the crystals under irradiation, the ion flux was kept below 1.8 × 10^8^ cm^−2^ s^−1^. The highest fluence here considered is 3.6 × 10^12^ cm^−2^, corresponding to a dose-equivalent field of 72 T. The thickness of all the investigated samples is lower than the longitudinal range of the ions into the material, that is about 14.5 *μ*m, as obtained by SRIM^48^ and PHITS^49^ code simulations, using the Kinchin-Pease approach. This guarantees that defects are introduced into the material without contributing extra charge. The overall damage can be computed by the mentioned codes, in terms of d.p.a. (displacements per atom, due to the elastic coulombian scattering against target nuclei), and of the total energy released by ionization. For the present experiment, we obtained the mean d.p.a. value of 7.6 × 10^−16^ × Φ, and a total energy release by ionization of 2.1 × 10^11^ × Φ eV cm^−3^, if the fluence Φ is expressed in cm^−2^.

### Measurements

In order to determine the critical temperature and the penetration depth we used a microwave resonator technique, in a cavity perturbation approach^38,39^. The resonator is made of a YBa_2_Cu_3_O_7−*x*_ film on MgO substrate, patterned in a coplanar geometry, with a 350-*μ*m-wide central stripline^50^. The Ba_1−*x*_K_*x*_Fe_2_As_2_ crystal is positioned by a small amount of high-vacuum grease in the center of the stripline, far from edges, i.e. in a region where the rf fields are uniform within about 5%. Measurements of the resonance curve are repeated in the same conditions, with and without the crystal, by means of a Rohde Schwarz ZVK vector network analyzer for an input power of −40 dBm, well below the non-linearity threshold for the resonator. The measurements were carried out in a Cryomech PT 415 pulse tube cooler.

The perturbations relative to no sample coupled to the resonator (rf field parallel to the broad face of the crystal), in terms of resonance frequency *f*_0_ and quality factor *Q*_0_ shifts, are^51^:

2 $$\frac{{\rm{\Delta }}{f}_{0}}{{f}_{0}}=\frac{1}{2}\frac{{V}_{s}}{{V}_{r}}\{1-{\Re }_{{\mathfrak{e}}}\,[\frac{\tanh (kc)}{kc}]\}$$

3 $${\rm{\Delta }}\,(\frac{1}{{Q}_{0}})=\frac{{V}_{s}}{{V}_{r}}{\Im }_{{\mathfrak{m}}}\,[\frac{\tanh (kc)}{kc}]$$

where *k* is the complex propagation constant and *c* is the crystal half thickness. Here *V*_*s*_ is the volume of the sample and *V*_*r*_ is the effective volume of the resonator. The geometrical factor (*V*_*s*_/*V*_*r*_) is determined in a self-consistent way from data above *T*_*c*_, where the crystals show a metallic behavior. In this case, ${\Re }_{{\mathfrak{e}}}(k)={\Im }_{{\mathfrak{m}}}(k)=1/\delta$ is imposed, where $\delta =\sqrt{2/\omega \mu \sigma }$ is the classical skin depth. Though most of the field penetrates through the broad faces of the crystals, suitable corrections are applied to account for the field penetration also along the sample thickness, due to the finiteness of the crystals (further details in the Supplementary Information). Once the geometrical factor is obtained, Eqs 2 and 3 allow obtaining the real and imaginary parts of the propagation constant *k* in the superconducting state, and in turn the London penetration depth *λ*_*L*_ and the normal conductivity *σ*_*n*_, that are related to *k* by^52^:

$$k={(\frac{1}{{\lambda }_{L}^{2}}+i\omega {\mu }_{0}{\sigma }_{n})}^{\frac{1}{2}}.$$

### The model

As mentioned above, we considered a three-band Eliashberg s± wave model in the presence of impurity scattering, by solving six coupled equations for the gaps Δ_*i*_(*iω*_*n*_) and the renormalization functions *Z*_*i*_(*iω*_*n*_), where *i* is a band index ranging from 1 to 3 and *ω*_*n*_ are the Matsubara frequencies.

The imaginary-axis equations^53–55^ read:

4 $${\omega }_{n}{Z}_{i}(i{\omega }_{n})={\omega }_{n}+\pi T\,\sum _{m,j}\,{{\rm{\Lambda }}}_{ij}^{Z}(i{\omega }_{n},i{\omega }_{m})\,{N}_{j}^{Z}(i{\omega }_{m})+\sum _{j}\,[{{\rm{\Gamma }}}_{{\rm{ij}}}^{N}+{{\rm{\Gamma }}}_{{\rm{ij}}}^{M}]\,{N}_{j}^{Z}(i{\omega }_{n})$$

5 $$\begin{array}{rcl}{Z}_{i}(i{\omega }_{n})\,{{\rm{\Delta }}}_{i}(i{\omega }_{n}) & = & \pi T\,\sum _{m,j}\,[{{\rm{\Lambda }}}_{ij}^{{\rm{\Delta }}}(i{\omega }_{n},i{\omega }_{m})-{\mu }_{ij}^{\ast }({\omega }_{c})]\,{\rm{\Theta }}({\omega }_{c}-|{\omega }_{m}|)\,{N}_{j}^{{\rm{\Delta }}}(i{\omega }_{m})\\  &  & +\sum _{j}\,[{{\rm{\Gamma }}}_{{\rm{ij}}}^{N}-{{\rm{\Gamma }}}_{{\rm{ij}}}^{M}]\,{N}_{j}^{{\rm{\Delta }}}(i{\omega }_{n})\end{array}$$

where ${{\rm{\Gamma }}}_{{\rm{ij}}}^{N}$ and ${{\rm{\Gamma }}}_{{\rm{ij}}}^{M}$ are the scattering rates from non-magnetic and magnetic impurities, respectively, ${{\rm{\Lambda }}}_{ij}^{Z}(i{\omega }_{n},i{\omega }_{m})={{\rm{\Lambda }}}_{ij}^{ph}(i{\omega }_{n},i{\omega }_{m})+{{\rm{\Lambda }}}_{ij}^{sf}(i{\omega }_{n},i{\omega }_{m})$ and ${{\rm{\Lambda }}}_{ij}^{{\rm{\Delta }}}(i{\omega }_{n},i{\omega }_{m})={{\rm{\Lambda }}}_{ij}^{ph}(i{\omega }_{n},i{\omega }_{m})-{{\rm{\Lambda }}}_{ij}^{sf}(i{\omega }_{n},i{\omega }_{m})$ where

$${{\rm{\Lambda }}}_{ij}^{ph,sf}\,(i{\omega }_{n},i{\omega }_{m})=2\,{\int }_{0}^{+\infty }\,d{\rm{\Omega }}{\rm{\Omega }}{\alpha }_{ij}^{2}{F}^{ph,sf}({\rm{\Omega }})/[{({\omega }_{n}-{\omega }_{m})}^{2}+{{\rm{\Omega }}}^{2}].$$

Θ is the Heaviside function and *ω*_*c*_ is a cutoff energy. The quantities ${\mu }_{ij}^{\ast }({\omega }_{{\rm{c}}})$ are the elements of the 3 × 3 Coulomb pseudopotential matrix. Finally, ${N}_{j}^{{\rm{\Delta }}}(i{\omega }_{m})={{\rm{\Delta }}}_{j}(i{\omega }_{m})/\sqrt{{\omega }_{m}^{2}+{{\rm{\Delta }}}_{j}^{2}(i{\omega }_{m})}$ and ${N}_{j}^{Z}(i{\omega }_{m})={\omega }_{m}/\sqrt{{\omega }_{m}^{2}+{{\rm{\Delta }}}_{j}^{2}(i{\omega }_{m})}$.

The electron-boson coupling constants are defined as ${\lambda }_{ij}^{ph,sf}=2\,{\int }_{0}^{+\infty }\,d{\rm{\Omega }}\tfrac{{\alpha }_{ij}^{2}{F}^{ph,sf}({\rm{\Omega }})}{{\rm{\Omega }}}$. The solution of Eqs 4 and 5 requires a huge number of input parameters that can be taken from literature or fixed by suitable approximations (further details in the Supplementary Information). Then the critical temperature can be calculated, which turns out to be in agreement with the experimental one, once the “feedback” effect^56^ of the electronic condensate on the antiferromagnetic spin fluctuations has been taken into account. The critical temperatures of the irradiated crystals are reproduced by setting a suitable non-zero interband scatting rate, as described above, in the *Results* section.

## Electronic supplementary material

Supplementary Info
