# Supplementary material for: Effects of disorder induced by heavy-ion irradiation on (Ba1−xKx)Fe2As2 single crystals, within the three-band Eliashberg s± wave model
Source: Sci Rep. 2017 Oct 12;7:13029. doi: 10.1038/s41598-017-13303-5 (PMC5638861; doi:10.1038/s41598-017-13303-5)
Supplement: Supplementary file 1 — Supplementary Info [file 41598_2017_13303_MOESM1_ESM.pdf]

Supplementary Information

Effects of disorder induced by heavy-ion irradiation on $(Ba_{1-x}K_x)Fe_2As_2$ single crystals, within the three-band Eliashberg s± wave model

G. Ghigo, G. A. Ummarino, L. Gozzelino, R. Gerbaldo, F. Laviano, D. Torsello, T. Tamegai

The three-band Eliashberg s± wave model

Parameter setting

As mentioned in the manuscript, the solution of the imaginary-axis Eliashberg equations requires a number of input parameters, i.e.:
i) the electron-phonon spectral functions $\alpha_{ij}^2 F^{ph}(\Omega)$;
ii) the electron-antiferromagnetic spin fluctuactions spectral functions, $\alpha_{ij}^2 F^{sf}(\Omega)$;
iii) the elements of the Coulomb pseudopotential matrix $\mu_{ij}^*(\omega_c)$;
iv) the nonmagnetic Γ_{ij}^N and paramagnetic Γ_{ij}^M impurity-scattering rates.

Some of these parameters can be found in literature and some others can be fixed by suitable approximations. In particular:

- as we refer to experimental data taken from single crystals of high quality, we can safely assume a negligible disorder for the unirradiated samples. The scattering rate from non-magnetic and magnetic impurities Γ_{ij}^N and Γ_{ij}^M can thus be taken to be zero for the unirradiated crystals. The way to account for non-negligible scattering rates in irradiated (disordered) crystals is discussed in the article.

- Following Mazin et al.[1], we can assume that:

a) the total electron-phonon coupling constant is small (the upper limit of the phonon coupling in the usual iron-arsenide compounds is ≈ 0.35)[2];
b) phonons do not contribute significantly to *inter*band coupling so that $\lambda_{ij}^{ph} \approx 0$. Moreover, the phonon contribution to *intra*band coupling is negligible, so that $\lambda_{ii}^{ph} \approx 0$, so as the Coulomb pseudopotential matrix: $\mu_{ii}^*(\omega_c) = \mu_{ij}^*(\omega_c) = 0$. [3–6];
c) spin fluctuations mainly provide *interband coupling between holes and electrons bands*, so that $\lambda_{ii}^{sf} \approx 0$;

Within these approximations, the electron-boson coupling-constant matrix λ_{ij} becomes:[3–5]

$$\lambda_{ij} = \begin{pmatrix} 0 & 0 & \lambda_{13}^{sf} \\ 0 & 0 & \lambda_{23}^{sf} \\ \lambda_{31}^{sf} = \lambda_{13}^{sf}\nu_{13} & \lambda_{32}^{sf} = \lambda_{23}^{sf}\nu_{23} & 0 \end{pmatrix} \tag{1}$$

where $\nu_{ij} = N_i(0)/N_j(0)$, and $N_i(0)$ is the normal density of states at the Fermi level for the i-th band. The coupling constants λ_{ij}^{sf} are defined through the electron-antiferromagnetic spin fluctuation spectral functions (Eliashberg functions) $\alpha_{ij}^2 F_{ij}^{sf}(\Omega)$. We choose these functions to have a Lorentzian shape:[3–5]

$$\alpha_{ij}^2 F_{ij}^{sf}(\Omega) = C_{ij}\big\{L(\Omega+\Omega_{ij},Y_{ij}) - L(\Omega-\Omega_{ij},Y_{ij})\big\},$$

where

$$L(\Omega \pm \Omega_{ij}, Y_{ij}) = \frac{1}{(\Omega \pm \Omega_{ij})^2 + Y_{ij}^2}$$

and C_{ij} are normalization constants, necessary to obtain the proper values of λ_{ij}, while Ω_{ij} and Y_{ij} are the peak energies and the half-widths of the Lorentzian functions, respectively (see fig.1).[6] In all the calculations we set $\Omega_{ij} = \Omega_0$, i.e. we assume that the characteristic energy of the spin fluctuations is a single quantity for all the coupling channels, and $Y_{ij} = \Omega_0/2$, based on the results of inelastic neutron scattering measurements.[7]

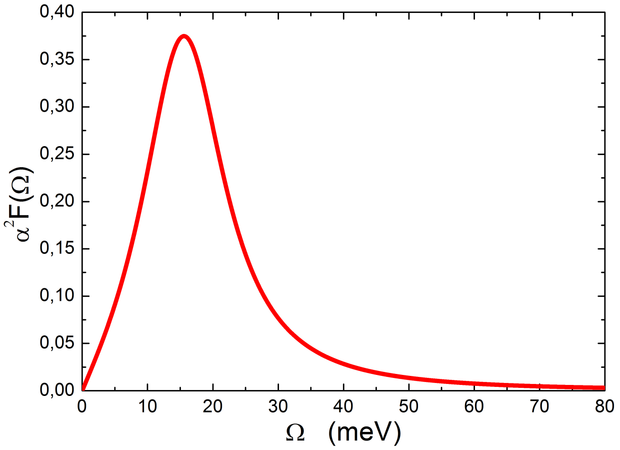

Figure 1. Antiferromagnetic spin fluctuactions spectral function normalized to λ=1.

• The peak energy of the Eliashberg functions, Ω_0, can be directly associated to the experimental critical temperature, T_c, by using the empirical law $\Omega_0 = 2T_c/5$ = 15.47 meV that has been demonstrated to hold, at least approximately, for iron pnictides.[8] We use a cut-off energy $\omega_c = 464$ meV and a maximum quasiparticle energy $\omega_{max} = 619$ meV. The factors ν_{ij} that enter the definition of λ_{ij} (eq.1) can be extracted from the ARPES measurements[9] by assuming that the Fermi momentum in the i-th band is proportional to the normal density of states at the Fermi level in the same band, i.e. $k_{Fi} \propto N_i(0)$. In this way, the ARPES results [6] lead to $\nu_{12} = 2$, $\nu_{13} = 1$ and $\nu_{23} = 0.5$.

Calculation of the energy gaps and T_c

• Now the model contains only two free parameters, λ_{13}^{sf} and λ_{23}^{sf}, and we want to reproduce the low-temperature gap amplitudes, which are actually obtained by analytical continuation of the imaginary solutions of the Eliashberg equations to the real axis by using the technique of the Padé approximants. Before irradiation, we find the values Δ_1=12.0 meV, Δ_2= 5.2 meV and Δ_3=$-$12.0 meV, in good agreement with earlier ARPES data. Actually, it turns out that in order to reproduce all the experimental gap values, the unique possibility is to set $\lambda_{13}^{sf} = 3.41$ and $\lambda_{23}^{sf} = 0.75$ for a total coupling $\lambda_{tot}^{sf} = 3.05$.

• Once all the parameters of the model have been fixed, we can calculate the critical temperature, that turns out to be equal to $T_c^* = 47.72$ K, while the experimental T_c is much lower (38.7 K for the unirradiated sample, if the temperature where the London penetration depth diverges is considered). However, we should still take into account the $feedback$ effect [6,10] of the electronic condensate on the antiferromagnetic spin fluctuations. To this aim, we consider the electron-boson spectral functions with an energy peak following the same temperature dependence of the superconductive gap ($\Omega(T) = \Omega_0 \tanh(1.76\sqrt{T_c^*/T - 1})$).[6] Of course, at $T = T_c^*$ the energy peak is equal to zero, while at T=0, the new spectral functions are equal to the old ones. This procedure leads to a critical temperature in agreement with the experimental one.

Microwave measurements

As mentioned in the manuscript, the geometrical factor (V_s/V_r) in Eqs. 2 and 3 is determined in a self-consistent way from data above T_c, where the crystals show a metallic behavior. In such conditions, $\mathfrak{Re}(k) = \mathfrak{Im}(k) = 1/\delta$, where $\delta = \sqrt{2/\omega\mu\sigma}$ is the classical skin depth. Moreover, the finite dimensions of the crystals has to be taken into account, resulting in the penetration of the field also from the lateral sides. This, in combination with Eqs. 2 and 3 gives:

$$2\frac{\Delta f_0}{f_0} = \frac{V_s}{V_r}\left[1 - A(a) - A(b) - A(c)\right]$$

$$\Delta\left(\frac{1}{Q_0}\right) = \frac{V_s}{V_r}\left[B(a) + B(b) + B(c)\right]$$

where

$$A(x) = \frac{\delta}{2x}\frac{\sinh\left(\frac{2x}{\delta}\right) + \sin\left(\frac{2x}{\delta}\right)}{\cosh\left(\frac{2x}{\delta}\right) + \cos\left(\frac{2x}{\delta}\right)}$$

$$B(x) = \frac{\delta}{2x}\frac{\sinh\left(\frac{2x}{\delta}\right) - \sin\left(\frac{2x}{\delta}\right)}{\cosh\left(\frac{2x}{\delta}\right) + \cos\left(\frac{2x}{\delta}\right)}$$

where $2c$ is the crystal thickness and $2a$ and $2b$ are the lateral dimensions of the sample. For such small crystals, it can be assumed that above T_c the temperature dependence of the shifts of both resonance frequency and quality factor is mainly due to the temperature dependence of the skin depth, and the small contribution given by the thermal expansion of the sample can be neglected. Figure 3 shows the experimental f and Q shifts fitted by the above equations, with the constraint to keep for both the same $\delta(T)$. The inset shows examples of direct measurements of resonance curves, at different temperatures, below and above T_c.

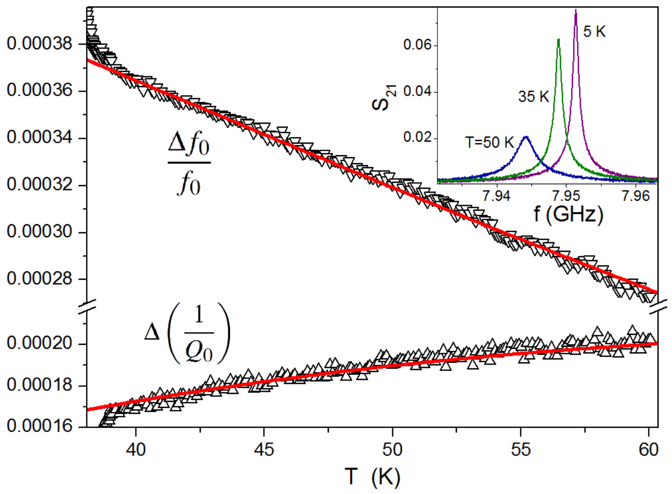

Figure 2. Shift of the resonance frequency and of the quality factor of the resonator due to the presence of the iron-based crystal under investigation. Data above T_c are fitted by the equations described in the text. Inset: examples of direct measurements at different temperatures, with the crystal coupled to the resonator

References

1. I.I. Mazin, D.J. Singh, M.D. Johannes, M.H. Du, Phys. Rev. Lett. 101, 057003 (2008)

2. L. Boeri, M. Calandra, I.I. Mazin, O.V. Dolgov, F. Mauri, Phys. Rev. B, 020506 (2010)

3. P.J. Hirschfeld, M.M. Korshunov, and I.I. Mazin, Rep. Prog. Phys. 74, 124508, (2011)

4. G.A. Ummarino, M. Tortello, D. Daghero, R.S. Gonnelli, Phys. Rev. B 80, 172503 (2009).

5. G.A. Ummarino, M. Tortello, D. Daghero, R.S. Gonnelli, J. Supercond. Nov. Magn. 24, 247, (2011)

6. G.A. Ummarino, Phys. Rev. B 83, 092508 (2011).

7. D.S. Inosov, J.T. Park, P. Bourges, D.L. Sun, Y. Sidis, A. Schneidewind, K. Hradil, D. Haug, C.T. Lin, B. Keimer and V. Hinkov, Nature Physics 6, 178 (2010).

8. Paglione and R.L. Greene, Nature Physics 6, 645 (2010)

9. H. Ding, P. Richard, K. Nakayama, K. Sugawara, T. Arakane, Y. Sekiba, A. Takayama, S. Souma, T. Sato, T. Takahashi, Z. Wang, X. Dai1, Z. Fang, G.F. Chen, J L. Luo and N.L. Wang, Europhys. Lett. 83, 47001 (2008).

10. A.V. Chubukov, D. Pines, and J. Schmalian, A Spin Fluctuation Model for d-Wave Superconductivity; D. Manske, I. Eremin, and K.H. Bennemann, Electronic Theory for Superconductivity in high-T_c Cuprates and Sr_2RuO_4, K.H. Bennemann and J.B. Ketterson Editors, Volume II. Superconductivity: Novel Superconductors, Springer-Verlag Berlin Heidelberg (2008).
